# Uncommon ascaris induced eosinophilic myocarditis simulating acute coronary syndrome without ST segment elevation: A case report

**DOI:** 10.1016/j.amsu.2022.103522

**Published:** 2022-03-28

**Authors:** Chaimae Toutai, Ilham Benahmed, Asmae Mrabet, Nabila Ismaili, Noha Elouafi

**Affiliations:** a Department of Cardiology, Mohammed VI University Hospital of Oujda, Mohammed First University of Oujda, Morocco; b Laboratory of Epidemiology, Clinical Research and Public Health, Faculty of Medicine and Pharmacy, Mohammed the First University of Oujda, Morocco

**Keywords:** *Ascaris lumbricoides*, Eosinophilic myocarditis, Acute coronary syndrome, Coronary angiography, Case report

## Abstract

**Introduction:**

and importance: *Ascaris lumbricoides* is a nematode parasite that causes ascariasis**.** Cardiac involvement in ascariasis is uncommon and rarely reported in the literature.

**Case presentation:**

We present the case of 45 years-old man, with previous medical history of ascaris infection one week before his admission to the emergency department for acute chest pain. The electrocardiogram revealed ST-segment and T wave abnormalities in septo-apico-lateral and inferior leads, along with high levels of troponin T and eosinophil blood cells count, while transthoracic echocardiography showed lateral and inferior walls motion abnormalities. The diagnosis of myocardial infarction was made and an urgent coronary angiography was carried out revealing normal coronary arteries which redressed the diagnosis and supported ascaris induced eosinophilic myocarditis (EM). The patient was put under anthelmintic drugs with favorable clinical, biological and imaging evolution.

**Clinical discussion:**

Eosinophilic myocarditis may present with variable and misleading scenarios ranging from asymptomatic patients to cardiogenic choc and sudden death and in some cases with clinical presentation of acute coronary syndrome.

**Conclusion:**

The aim of this work was to increase recognition of EM in the light of this clinical case report of ascaris lumbricoides associated myocarditis simulating an acute coronary syndrome without ST segment elevation.

## Highlights

• Eosinophilic myocarditis is a rarely reported and underestimated condition.
• It is difficult to differentiate eosinophilic myocarditis from acute myocardial infarction.
• The diagnosis of EM requires the association of a set of arguments.
• The rapid initiation of adapted treatment based on anthelmintic drugs and corticosteroids is necessary to avoid fatal course of this disease.

## 1 Introduction

Eosinophilic myocarditis (EM) is a rarely reported and underestimated condition, with no clear diagnosis criterion neither management guidelines this far. It may present with variable and misleading scenarios. The aim of this work was to increase recognition of EM in the light of this clinical case report of ascaris lumbricoides associated myocarditis simulating an acute coronary syndrome without ST segment elevation. The subsequent diagnostic evaluation, the possible mechanisms underlying this association and the management of this condition will be discussed in this case report. This work has been reported in line with THE SCARE 2020 criteria [12].

## 2 Patient information

**Fig. 1:**
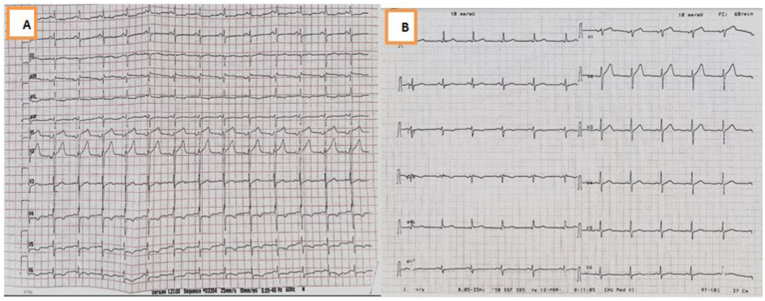
Electrocardiogram findings of the patient. A: Electrocardiogram on admission shows alterations of repolarization in septo-apico-lateral and inferior leads B: Normalization of Electrocardiogram after 3 months.

**Fig. 2:**
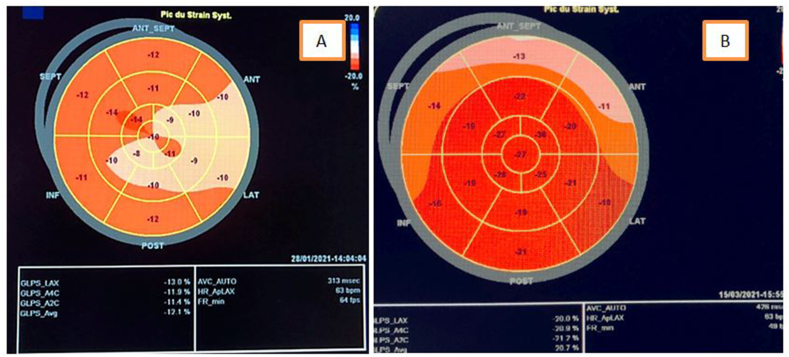
Electrocardiography findings A: Electrocardiography on admission shows strain alteration in the lateral and inferior walls B: Electrocardiography shows strain recovery after 3 months.

**Fig. 3:**
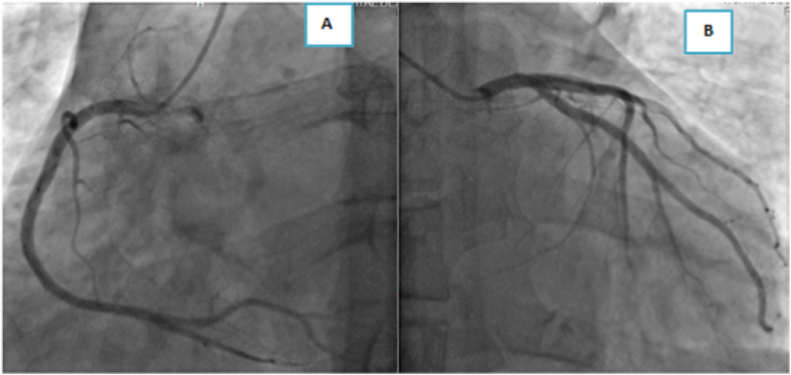
Coronary angiography upon admission. A: Angiographically normal right coronary system B: Angiographically normal left coronary system.

We report the case of a 45 -years-old man, without any predisposing risk factor for coronary artery disease neither particular family history or psychosocial history. The patient has been followed for seven years for indeterminate colitis revealed by chronic abdominal pain and sub occlusive syndrome with signs of chronic inflammatory bowel disease not corresponding to ulcerative colitis or crohn's disease in the colon and gastrointestinal biopsy specimens. The patient was put under symptomatic treatment of abdominal pain without any clinical improvement. The patient was admitted to our hospital complaining of intense chest pain of 4 day's duration with abdominal pain and sub occlusive syndrome. Through questioning elicited the history of hospitalization, one week before his admission, for the management of acute aggravation of abdominal pain with diarrhea related to ascaris lumbricoides infection detected by stool examination and treated by albendazole 400 mg as a single dose. No history of allergic disease or recent traveling or drug usage was found. On admission, the patient had normal blood pressure at 110/70 mm Hg, pulse was 90 beats/min, respiratory rate was 18/min, and body temperature was 36.8 °C. Physical examination revealed diffuse abdominal sensibility and a pruritic rash over his lower limbs.The initial electrocardiogram, in comparison with a previous one that was within normal limits, objectified a regular sinus rhythm with ST-segment depression in septo-apico-lateral leads and negative T waves in the same territory and steep ST in the inferior leads (Fig. 1,A). Transthoracic echocardiography (TTE) showed a non-dilated, non-hypertrophied left ventricle, with hypokinesis of lateral and inferior walls with preserved ejection fraction of 57% without any pericardial effusion (Fig. 2, A). Troponin T level was elevated at 6000 ng/l, reference value being less than 26 ng/l. The patient was admitted to the intensive care unit of cardiology with the diagnosis of acute non ST elevation myocardial infarction (NSTEMI), thus a coronary angiography was carried out revealing normal coronary arteries (Fig. 3A and B). Initial laboratory investigations revealed an inflammatory syndrome; CRP at 100 mg/L, white blood cells count at 28060/mm3 with 24,9% neutrophils, 7,3% lymphocytes, 3,8% monocytes, and 63,6% eosinophil's corresponding with an absolute eosinophil count of 17840/mm3 (normal range, 0–500/mm3) and platelets count was 256000/mm3. A complete 3 stool samples analysis detected ascaris lambricoid eggs. Tests for antinuclear antibody (ANA) and antineutrophil cytoplasmic antibody (ANCA) were negative. Serologies for human immunodeficiency virus, viral hepatitis and syphilis were negative along with positive IgG but negative IgM antibodies for both cytomegalovirus and toxoplasmosis. Therefore, the diagnosis of ascaris associated eosinophilic myocarditis was retained on the basis of clinical presentation, ECG and echocardiography findings with elevated troponins and normal coronary arteries associated with eosinophilia due to a large infestation by ascaris lumbricoides. Thoracic and abdominal computed tomography showed a stable appearance of digestive inflammatory thickening with submucosal edema of the right colon, duodenum and the last ileal loop with regular symmetrical and circumferential thickening of the lower esophagus. In addition, the CT scan also revealed a low-abundance right pleural effusion along with right basal alveolar-interstitial lung disease (loeffler's syndrome).

The patient was treated with albendazole 400 mg for 4 days and bisoprolol 5 mg for ventricular excitability revealed in ECG monitoring. Within one week of treatment, the patient's clinical symptoms improved, he reports less abdominal pain without chest pain recurrence. The leukocyte count decreased from 28060/mm3 to 14770/mm3 and the eosinophil count from 64 to 61%.

After three months, the control of ECG and echocardiography revealed no repolarization or segmental kinetics abnormalities with normalization of leukocyte count at 6670/mm3, eosinophilic count also remarkably decreased to 1307/mm3 corresponding to 19% of white blood cells (Fig. 1,B) (Fig. 2,B).

## 3 Discussion

Eosinophilic myocarditis (EM) is a relatively rare form of myocarditis in all age groups, characterized by diffuse or focal myocardial inflammation with eosinophilic infiltration, generally associated with high levels of peripheral eosinophilic cells count [1]. A definitive diagnosis of eosinophilic myocarditis requires association of two criteria, first of all, fulfillment of the four criteria for diagnosis of myocarditis (ECG or Holter or stress test features, elevated myocardiocytolysis markers (troponin I or T), functional and structural abnormalities in cardiac imaging and tissue characterization on cardiac magnetic resonance as well as histological diagnosis based on the presence of inflammatory eosinophilic cell infiltrate [2]. The degree of eosinophilic infiltration of the myocardium depends on the underlying cause and the degree and duration of eosinophilic exposure.

Eosinophilic myocarditis has a male predilection with the mean age at presentation ranges from 20 to 51 years and some pediatric cases have been reported also [3]. Cardiac involvement in eosinophilia occurs in 54–82% of cases [4], it was first reported in 1936 by Loffler who described the observation of "endocarditis parietalis fibroplastica " associated with blood eosinophilia [5], while myocardial and vascular damage due to eosinophilic infiltration is rarely reported and diagnosed during life because of the nonspecificity of the clinical manifestations which delays diagnosis and the rapidly fatal course of eosinophilic arteritis and myocarditis, which are usually diagnosed on postmortem.

Eosinophilic myocarditis has been reported in association with several conditions including idiopathic hypereosinophilic syndrome (HES) when no cause is identified, Loeffler endocarditis, tropical endomyocardial fibrosis and vasculitis such Churg-Strauss syndrome known as eosinophilic granuloamtosis with polyangiitis (EGPA). Other etiologies include infections, allergic disease, allograft rejection in heart transplantation and some malignancies especially myeloproliferative syndromes as well as hypersensitivity myocarditis which is the most common cause reported [1]. In our patient even if eosinophilia was clearly associated to ascaris infection supported by stool examination, a systematic evaluation was made including the research of drug usage and specific testing for the common causes of hypereosinophilia and myocarditis mentioned earlier, eliminating thus any viral infection, or malignancies nor vasculitis.

Generally, parasitic infestation is the commonest identifiable cause of eosinophilia. Ascaris. lumbricoides is the most common parasitic infection of humans. Most persons infected with ascaris lumbricoides infection are asymptomatic while a small proportion of patients develop pulmonary symptoms including nonproductive cough, chest discomfort, fever and dyspnea to eosinophilic pneumonia (Loeffler syndrome) in severe cases. Myocarditis in ascariasis can occur from direct larval invasion of the myocardium or from reactive eosinophilia that develops in the first three weeks when larvae invade the lung tissue and provoke an immune-mediated hypersensitivity response. In severe reactive eosinophilia, infiltration of several tissues may occur including the lung, heart, kidneys, bone marrow, gastrointestinal tract and gallbladder [6].

The proposed mechanisms of cardiac injuries related to high eosinophilic cells count evolves in three stages. The first stage being the acute necrotic stage as in our patient. During this stage, most patients are asymptomatic and myocarditis is characterized by inflammation, eosinophilic infiltration of the myocardium and in some case the development of eosinophilic granulomas which degranulation release eosinophilic cytotoxic proteins such as eosinophilic cationic proteins, major basic protein, eosinophil-derived neurotoxin, and eosinophil peroxidase as well as reactive oxygen species, leading to myocyte mitochondria dysfunction, endomyocardial necrosis and apoptosis. Moreover, Eosinophil granule proteins are also major potent procoagulant factors responsible of platelets stimulation and impairment of the anticoagulant properties of endothelial membrane leading to the second and thrombotic stage, known as the mural thrombus stage, characterized by the formation of intracardiac mural thrombi that may lead to thromboembolic events. The third and final stage is the fibrotic stage characterized by fibrosis of the thrombus-damaged myocardium. This eventually leads to restrictive cardiomyopathy as well as atrioventricular valve dysfunction [3].

Eosinophilic myocarditis can present as a chronic or acute condition. Acute necrotizing myocarditis (NEC) is the initial presentation of this disease, it's an aggressive form that follows a rapidly deteriorating cardiac course with less systemic involvement, while chronic myocarditis ranges mostly from one to 11 years from diagnosis symptoms and patients present mostly with symptoms of congestive heart failure [3].

The spectrum of presentations of EM is variable and the clinical severity does not correspond with the level of peripheral eosinophilia. Patients may present with fever, skin rash, palpitations, chest pain, shortness of breath, and symptoms of heart failure to cardiogenic shock. Moreover, conduction delays, ST and T abnormalities and cardiac arrhythmias have also been reported. Arterial embolism, pericarditis and sudden death are possible presentations as well. Last but not least, EM was also associated with myocardial infarction and several mechanism were incriminated including coronary stenosis secondary to coronary arteritis, left descendent artery dissection and coronary vasospasm due to eosinophilic proteins and vasoactive cytokines that stimulate constriction of vascular smooth muscle [3,7].

In some cases, it is difficult to differentiate myocarditis from acute myocardial infarction due to similar presentations, thus, the diversity and non-specificity of clinical presentations of EM and the lack of diagnostic criterion guidelines implies that the diagnosis requires a high level of clinical suspicion and the association of a set of arguments and investigations. First of all; medical history of the patient should always be reviewed especially history of allergic or systemic disease as well as history of infection, traveling or any drug usage before the onset of symptoms. Laboratory findings are also crucial; in fact**,** the increased level of eosinophil cells count, although not specific and not present in all cases, supports the diagnosis of eosinophilic myocarditis, and some studies have demonstrated that the number of degranulated eosinophil's and serum eosinophilic cationic proteins (ECP) levels can be valuable parameters for diagnosis and monitoring of disease activity under treatment [8]. Furthermore, inflammatory markers, cardiac troponin I and T levels are often increased in EM however they are non-specific and when normal do not exclude the diagnosis [2]. Echocardiographic findings of EM are variable, they depend on the stage of evolution of EM and may present as dilated, hypertrophic, restrictive or ischemic cardiomyopathy. Endocardial thickening, intracardiac thrombi, pericardial effusion, diastolic dysfunction, global systolic ventricular dysfunction and regional wall motion abnormalities have been described. Our patient had normal left ventricular size and global systolic function with regional wall motion abnormalities. Echocardiographic features have been shown to be reversible with therapy, as seen in our case [2,3,9]**.** In addition, coronary angiography is mandatory to exhibit coronary artery disease such as stenosis, arteritis or thrombosis [2]. Cardiovascular magnetic resonance (CMR) provides crucial information concerning myocardial tissue characterization, myocardial perfusion, ventricular function and fibrosis and remains the only noninvasive method that can be used prior to endomyocardial biopsy (EMB) to support the diagnosis of myocarditis, to visualize the extent of endomyocardial involvement as well as to monitor disease progression during treatment. However, even if CMR imaging is more sensitive and specific than EMB, it can't provide information about the degree of inflammation and the etiology of myocarditis [2,3].

Nuclear imaging is not routinely recommended for the diagnosis of myocarditis, because of its limited availability and risk of radiation exposure [2]. EMB employing Dallas criterion has been considered the gold standard for diagnosis and identifying the etiology and type of myocarditis based on the cellular infiltrate. In eosinophilic myocarditis, EMB reveals diffuse myocardial necrosis in association with eosinophilic infiltration and fibrosis of the myocardial interstitium along with focal myocyte dissolution and perivascular infiltration [4]. However, despites its grate value, EMB remains an invasive tool that can't be performed in all patients and implies an experienced team, furthermore, it has a limited sensitivity of 50% and specificity because the infiltrates are often local and may expose also to the risk of embolic events in the presence of intra-cardiac thrombi.

Ascaris induced EM has been reported in two cases until now. In the first case, the patient presented with symptoms resembling an acute myocardial infarction with ST segment elevation and cardiogenic choc [9], in the second case report, the patient was asymptomatic but TTE revealed a dilated and hypertrophic left ventricle (LV) with apical endocardial thickening, diffuse hypokinesis and reduced LV ejection fraction of 47% [6]. Herein we present an exceptional case of ascaris-induced EM presenting with symptoms mimicking an acute myocardial infarction without ST segment elevation and preserved LV global systolic function. In our patient, the diagnosis of EM was made in the presence of acute chest pain in a patient with history of ascaris infection one week prior admission, without predisposing risk factors for coronary heart disease associated with ischemic ECG changes, marked elevation in myocardial enzymes, along with left ventricular regional wall motion abnormalities and normal coronary arteries. This diagnosis was supported by the presence of marked peripheral eosinophilia and stool examination positive for ascaris lumbricoides. The rapid diagnosis of EM was made allowing timely and appropriate therapy with albendazole that improved clinical manifestations and biological findings along with normalization of electrocardiogram and echocardiographic features. In our patient neither myocardial biopsy nor CMR imaging were performed because the diagnosis was clear and because the patient did not afford doing these investigations.

There are no clear guidelines or consensus concerning the treatment of EM. Management of this disease depends on the underling etiology, clinical presentation and the stage of the disease and consists of withdrawal of the possible offending agents, standard treatment for heart failure and early treatment with high doses of steroids [3]. Initiation of steroids in the early stage of EM can achieve drastic improvements in clinical outcomes and prognosis and may prevent progression of cardiac damage [3]. Corticosteroids were found to be used in 77,7% of 10.13039/100006138EM related to systemic diseases such as EGPA and HES and less frequently in the hypersensitivity group (68,8%) with a complete recovery and normalization of cardiac contractility after treatment in many patients which supports the usefulness of corticosteroids [1]. There are no clinical trials that tested the efficiency of steroids in parasitic eosinophilic myocarditis, however the usage of anthelminthic agents is strongly recommended and should be administrated early in the clinical course. In fact, In 3 case reports of EM related to parasitic infection, Ascarisis (Ascaris lumbricoidis), Trichinellosis (*Trichinella spiralis*) and Visceral larva migrans (Toxocara canis) respectively the patients were treated by dual therapy associating albendazole and high doses of prednisolone with gradual tapering with favorable outcomes [[9], [10], [11]]. In our case report as in the case of Pott-Junior and Ferraz, the patients were treated only by albendazole with favorable outcomes as well [6].

The role of immunosuppressive therapy is yet controversial and immunosuppressive drugs may prevent recurrences. Treatment with cytotoxic agents such as cyclophosphamide and imatinib may be used as a specific treatment in EM associated with eosinophilic granuloamtosis with polyangiitis (EGPA) or myeloprolifertive syndromes. Recently, mepolizumab represents an emerging strategy targeting interleukin-5 (IL-5) using a humanized blocking monoclonal antibody and in consequence reducing and stabilizing blood eosinophil count which is the target for patients with EM associated to idiopathic hypereosinophilic syndrome (HES) to prevent long-term corticosteroid therapy undesirable side effects [1]. Inotropic agents and left ventricular assist device (LVAD) support are very useful in hemodynamically unstable patients and for refractory conditions in cases of heart failure or arrhythmia [1]. The role of anticoagulation during acute EM for preventing endocavitary thrombi and arterial embolism was discussed but no study proved the efficiency of preventive anticoagulation therapy.

Eosinophilic myocarditis is a rare, fatal and under recognized disease frequently diagnosed in the post mortem examination. In the acute necrotizing form, the evolution is quite often fatal due to acute heart failure and cardiogenic choc in the fulminant form and if not treated, cardiac damage progress leading to the thrombotic and fibrotic stages and there complications ranging from restrictive cardiomyopathy and intracavitary thrombi to life threatening arrhythmias and arterial embolism. The real mortality rate of EM remains unknown, brubati and al. reports an intra-hospital mortality of 17% while the five**-**year mortality was about 30% [2].

## 4 Conclusion

Eosinophilic myocarditis remains a very rare and underestimated condition, most frequently diagnosed on post mortem autopsies. In our patient, the history of previous ascaris infection and increased blood eosinophilic cells count aroused the diagnosis of eosinophilic myocarditis. This case report emphasizes the importance of a careful questioning and high index of clinical suspicion of EM on admission of any patient presenting for acute coronary syndrome with normal coronary arteries, especially in the presence of eosinophilia, allowing thus, the rapid initiation of adapted treatment to avoid rapid and often fatal course of this disease. The lack of clear guidelines concerning diagnosis and treatment of eosinophilic myocarditis requires large and randomized studies to be carried out to establish diagnosis criterion and standard management guidelines to improve the prognosis of theses disease.

## Ethical approval

Not required for this case report.

## Sources of funding

This research did not receive any specific grant from funding agencies in the public, commercial, or not-for-profit sectors.

## Author contributions

Chaimae Toutai wrote the manuscript and conducted the literature review. Ilham Benahmed and Asmae Mrabet provided data collection. Nabila Ismaili provided supervision and data validation. Noha El Ouafi provided supervision and data validation. All the authors approved the final draft of the paper, for the submission.

## Trail registry number

This is not an original research project involving human participants in an interventional or an observational study but a case report. This registration is not applicable.

## Guarantor

Chaimae Toutai.

## Consent

Written informed consent was obtained from the patient for publication of this case report and accompanying images. A copy of the written consent is available for review by the Editor-in-Chief of this journal on request.

## Provenance and peer review

Not commissioned, externally peer-reviewed.

## Declaration of competing interest

There are no conflicts of interest.
